# Application of Spectroscopy in Additive Manufacturing

**DOI:** 10.3390/ma14010203

**Published:** 2021-01-04

**Authors:** Jingjunjiao Long, Ashveen Nand, Sudip Ray

**Affiliations:** 1 Orthopedic Research Institute, Department of Orthopedics, West China Hospital, Sichuan University, Chengdu 610041, China; 2 School of Environmental and Animal Sciences and School of Healthcare and Social Practice, Unitec Institute of Technology, Auckland 1025, New Zealand; 3 MBIE ^NZ^Product Accelerator Programme, School of Chemical Sciences, University of Auckland, Auckland 1010, New Zealand

**Keywords:** 3D-printing, additive manufacturing, spectroscopy

## Abstract

Additive manufacturing (AM) is a rapidly expanding material production technique that brings new opportunities in various fields as it enables fast and low-cost prototyping as well as easy customisation. However, it is still hindered by raw material selection, processing defects and final product assessment/adjustment in pre-, in- and post-processing stages. Spectroscopic techniques offer suitable inspection, diagnosis and product trouble-shooting at each stage of AM processing. This review outlines the limitations in AM processes and the prospective role of spectroscopy in addressing these challenges. An overview on the principles and applications of AM techniques is presented, followed by the principles of spectroscopic techniques involved in AM and their applications in assessing additively manufactured parts.

## 1. Introduction

Additive manufacturing (AM), commonly known as 3D printing, is a process of fabricating 3-dimensional objects through layer-by-layer addition, directly from a digital file. The AM technology encompasses a range of production techniques such as fused deposition modelling (FDM), powder bed fusion, inkjet printing, stereolithography (SLA) and laser engineered net shaping (LENS). AM presented a market size of USD 7.97 billion in 2018 with a compound annual growth rate (CAGR) of 14.4%, expecting to reach USD 23.33 billion by 2026 [1]. The fast-growing interests and evolutions in AM will boost its market uptake and commercial chance. AM has been employed in construction [2], biomechanical [3], healthcare [4], aerospace [5] and automotive industries [6] and with advancements in AM techniques, new applications keep emerging. A wide variety of materials, in the likes of metals, alloys, ceramics and polymers, have been used in AM applications. Some examples of recent AM developments having commercial implications are presented in Table 1.

**Figure 1:**
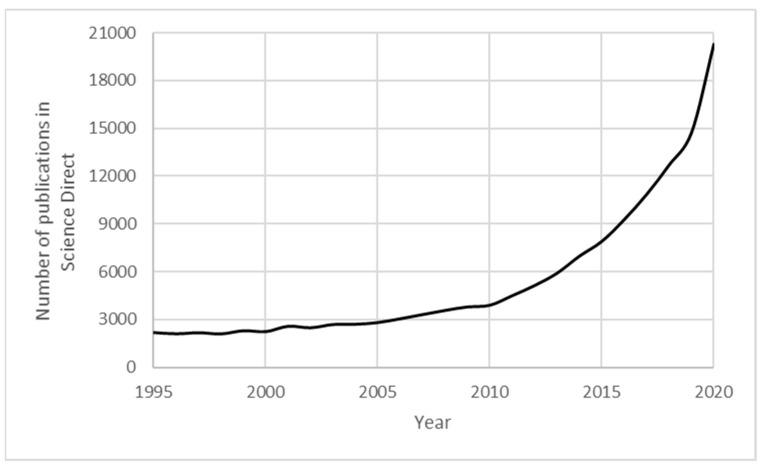
Growth of interest in additive manufacturing. (Source: ScienceDirect; keywords: additive manufacturing).

The concept of creating 3D objects in a layer-by-layer fashion using computer-aided designs, then commonly known as rapid prototyping, was born in the 1980s. Rapid prototyping was traditionally used to create models to realise concepts physically [12]. However, AM has now evolved from a prototyping tool to a production technique for actual functioning products [13,14]. The growth in popularity of AM, as shown in Figure 1, is attributed to its advantages such as; fabrication of complex geometries with high precision in a short period of time, operative with very little human intervention and reduced material wastage [15,16,17]. 

While AM is gaining popularity, one of the major challenges has been the lack of understanding of the fundamental material properties of produced parts and raw materials used. Non-destructive techniques such as thermography and acoustic emission testing have been applied to detect defects in additively manufactured parts [18]. Raw materials have been characterised using laser particle size distribution measurements, X-ray computed tomography, X-ray diffraction, scanning electron microscopy, dispersive elemental analysis [13]. However, these techniques have fallen short of providing information on the chemistry of the materials. Spectroscopic material characterisation techniques, though currently used sparingly in AM, have the potential to bridge the knowledge gap in material properties for AM. Spectroscopic techniques offer rapid analysis with a high degree of sensitivity. Most spectroscopic techniques find use in real-time production process monitoring for quality control purposes without the need for withdrawing materials from the production line. Spectroscopic analysis provides insights into structures at molecular level and reveals the chemical composition and composition variation of the material. Understanding the material properties and physical behaviours, leads to development of innovative materials, customised products and prolonged shelf life of the final products. Knowledge of molecular structure helps material scientists and engineers to choose the correct materials for specific functional properties in end products. Moreover, this will also help in reducing material waste and enhance production efficiency.

Spectroscopic techniques can be utilised for raw material characterisation for quality control purposes, preventing wastage and economical loss as well as superior manufactured products. Moreover, characterisation during the manufacturing process would ensure control of the process so that parts are manufactured as planned. Dispersion of additives in the matrices, chemical bonding between different components and presence of functional materials can be verified using spectroscopy.

4D printing, which is based on 3D printing but using stimulus-responsive materials to change its property, shape or character as a function of time, has been attracting intense attention since 2013 when it was introduced by a research group at MIT [19]. However, as an emerging technique, the products from 4D printing are mostly developed as conceptional objects. There is a huge gap to transfer great concepts into practical products in 4D printing, where real-time monitoring and control should be industrialised. For example, Shiblee et al. [20] reported a 4D printed flower structure with swelling-induced ‘line-to-coil’ bending, showing great potential to function as biomimetic actuators, encapsulating systems and soft robotic. However, refined control of the curvature over time remains to be conquered before turning into industrial products, where real-time monitoring and in-time feedback is needed. Understanding the time-dependant molecular change would make a great contribution towards further development of 4D printing products with refined control of the changes over time. Hence, spectroscopy finds its great application to achieve real-time and in situ detection with time changing in 4D printing. 

This paper aims to provide a comprehensive review of spectroscopic techniques currently employed and future prospective as material characterisation tools in AM. The specific aims of this review are: (i) To summarise the principles, production techniques and materials used in AM; (ii) to provide the principles of spectroscopic techniques; (iii) to outline the scientific and technical challenges in AM and the potential functionality of spectroscopy in the AM process.

## 2. AM Techniques

**Figure 2:**
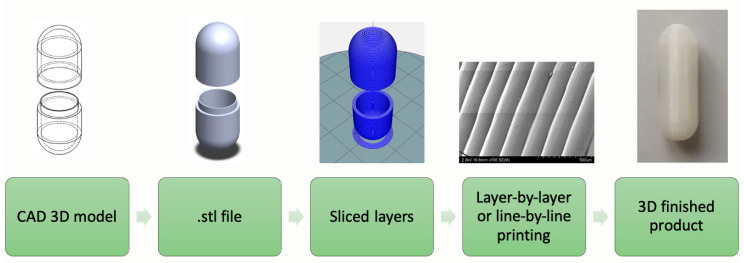
Flow diagram of additive manufacturing (AM) process.

Unlike traditional subtractive manufacturing processes such as lathing, milling or grinding, AM produces desired shapes by addition of materials without any part specific tooling [15,18]. There are a range of AM techniques available, however, all work on the same principle are shown in Figure 2. The design of the part to be fabricated is created by computer-aided design (CAD) software, which is then converted to standard tessellation language (.stl) file. The .stl file then slices the 3D model into layers and the AM apparatus fabricates the part by accumulation of successive layers shaped in x-y plane. The assembly of single layers on top of each other brings about the z dimension, hence a 3D object [17]. 

A range of AM techniques, working in different ways, are currently available [12,13,15,16,17]. The major AM techniques summarised and tabulated in Table 2, work by either thermoplastic filament extrusion, photo polymerisation, melt deposition, ceramic suspension deposition or lamination. The choice of the technique depends on the raw material used and end product fabrication needs. In order to realise the importance of material characterisation, it is imperative to understand how different AM techniques work.

### 2.1. Fused Deposition Modelling (FDM)

**Figure 3:**
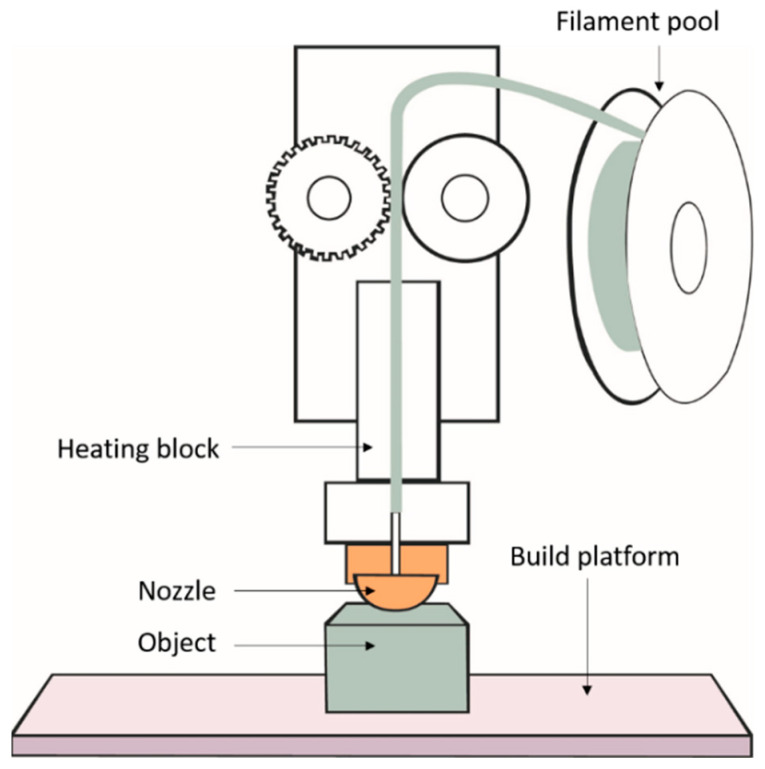
Schematic illustration of fused deposition modelling (FDM) system. Reproduced from [23], with permission from Eureka Science (FZC) conveyed through Copyright Clearance Center, Inc.

In FDM, a thermoplastic filament is fed to the heated printer head and molten polymer is extruded through the nozzle to create a thin layer of the desired shape on a platform. The printer head moves according to a programmed mechanism and deposits another thin layer on the earlier printed layer. Successive layers fuse together and solidify at room temperature to result in the desired 3D part. The FDM technology has been developed further to make use of two extrusion nozzles. While one nozzle extrudes the thermoplastic to build the part, the second nozzle dispenses material for the creation of a sacrificial support structure which can be removed after printing [21]. This development has paved way for printing overhanging geometries [22]. The schematic process of FDM is presented in Figure 3.

FDM is favoured due to its low cost, high speed and no post processing chemical curing [12,16]. Its major disadvantage, however, is mechanical weakness of the printed part due to inter-layer distortions [24]. The layer-by-layer appearance also projects poor quality surface finish of the produced part [25]. 

### 2.2. Powder Bed Fusion

**Figure 4:**
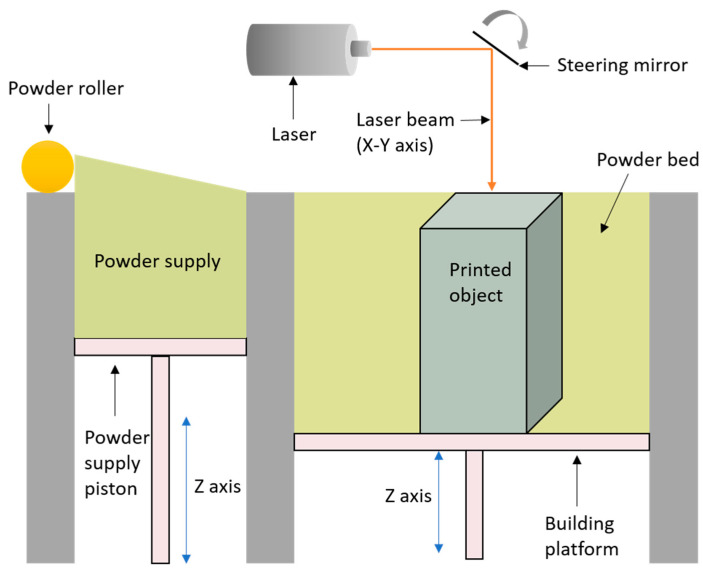
Schematic illustration of powder bed fusion system (SLS).

Powder bed fusion is an AM technique where powder material is sintered or fused together using a laser beam. A thin layer (0.1 mm) of the material powder is spread on a platform and the laser fuses the powder at specific locations specified by the design [12]. New layer of the material powder is spread across the previous layer using a roller and then fused together. The layering and fusion of the powder is repeated until the desired 3D object is made [16]. Selective laser sintering (SLS) and selective laser melting (SLM) are the two aspects of powder bed fusion technique. SLS achieves fusion of material powders by elevating local temperature on the surface of the grains without melting them completely and can be applied to a wide range of polymers, metals and alloys. SLM, on the other hand, fuses the material powders by completely melting them and is used for only certain metals such as aluminium and steel. Parts formed by SLM have superior mechanical properties [16,26]. The schematic process of powder bed fusion is presented in Figure 4.

Powder bed fusion attracts attention as it could be used with a wide range of material powders such as polymers, metals, ceramics and their combinations [17,27]. Moreover, the powder bed itself provides support for the printed part so additional efforts of removing the support material is eliminated [16]. However, accuracy is limited by the particle size of the material powder and an inert gas atmosphere has to be maintained for the process to prevent oxidation [12]. Additionally, some critical drawbacks are attributed to powder bed fusion, such as undesired porosity and balling, residual stresses, cracks or layer delamination and microstructure inhomogeneity. 

### 2.3. Laser Engineered Net Shaping (LENS)

**Figure 5:**
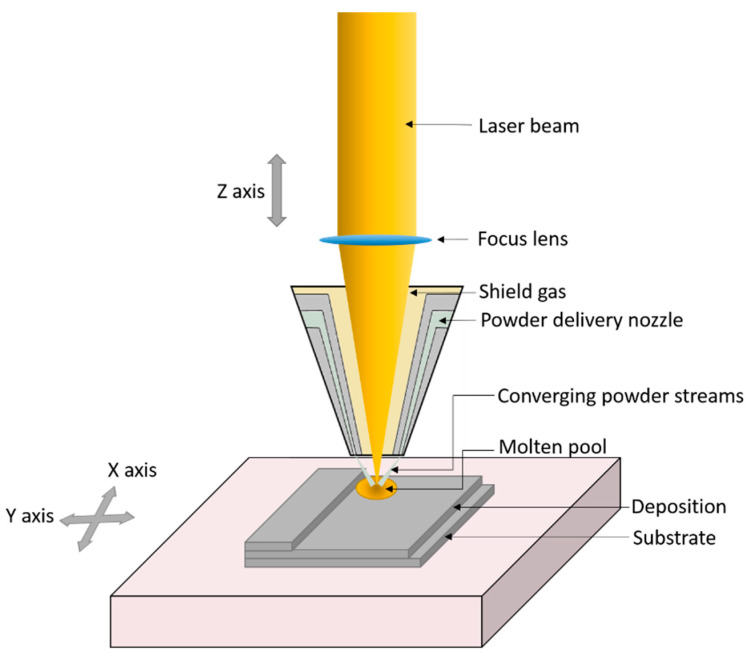
Schematic illustration of laser engineered net shaping (LENS) system.

In LENS, molten metal powder, with particle size ranging from 38–150 µm, is injected onto a specific location on a substrate stationed on a base plate. A strong laser is used to melt the metal powder and a melt pool is created on the substrate. The deposition happens in the X − Y direction to fabricate layers in an additive manner [27]. The process occurs in a closed chamber flushed with argon and the printed layer solidifies as it cools. LENS differs from SLS and SLM as it does not use a material powder bed but the metal powder is fed directly to the processing apparatus. LENS can be helpful in filling cracks or repairing broken parts [12,16]. The schematic process of LENS system is presented in Figure 5.

LENS permits the use of a large range of metals and is used to repair parts which would otherwise have been impossible or very expensive. While material graded deposition allows for more than one type of metal powder to be used simultaneously, the gradient material offers enhanced compatibility and high mechanical properties equivalent to wrought processing for many applications. The disadvantage of LENS, however, is the residual stress created on the parts by the uneven heating and cooling [12]. 

### 2.4. Laminated Object Manufacturing (LOM)

**Figure 6:**
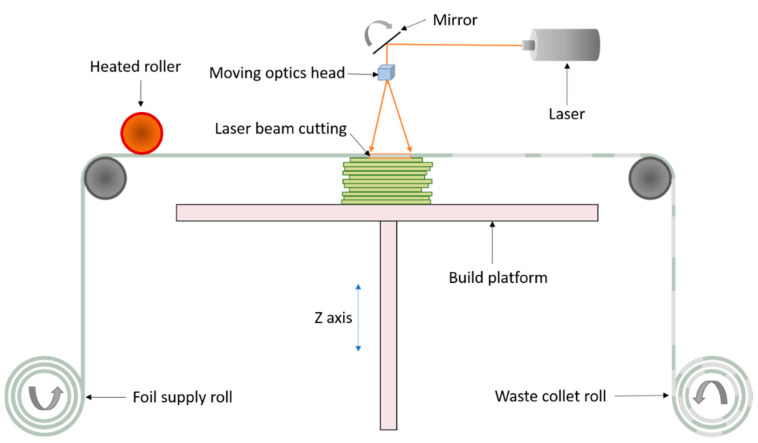
Schematic illustration of laminated object manufacturing (LOM) system.

The LOM technique uses a carbon dioxide laser to cut modelled shapes from a thin sheet of material fed into the machine. Successive layers of precisely cut shapes or laminates are bonded together by application of pressure, heat and a thermal adhesive [12,16]. The excess material left after cutting are usually removed and recycled. The schematic process of LOM system is presented in Figure 6. LOM is a low-cost process with no post processing requirements. LOM also presents an excellent technique for manufacturing large structures. On the other hand, the drawbacks of LOM are low surface definition and difficulty in building complex internal cavities [12,16].

### 2.5. Stereolithography (SLA)

**Figure 7:**
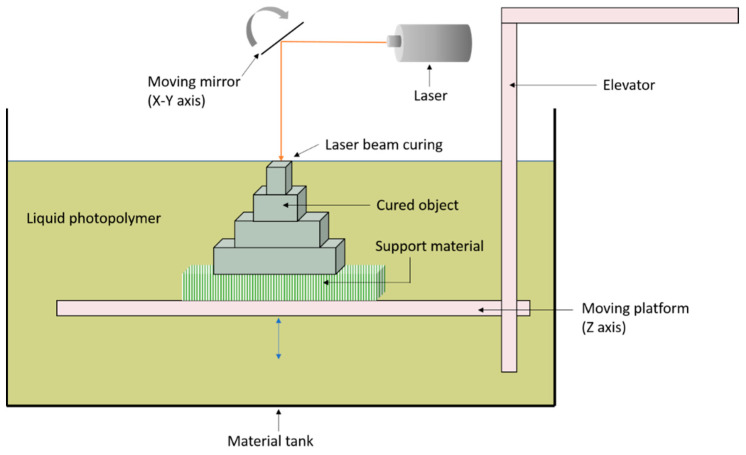
Schematic illustration of stereolithography (SLA) system.

Developed in the late 1980s, SLA is one of the earliest AM techniques [28]. It is based on the concept of photopolymerisation of an UV active monomer. The SLA technique involves lowering a build platform into a monomer reservoir, coating the platform with a usually 50–75 µm thick layer of liquid monomer [13]. A laser beam focuses on the liquid surface and draws out the shape according to the CAD model. UV irradiation is absorbed by the photo-initiator which activates the polymerisation of the monomer, transforming the liquid monomer to a solid polymer. The build platform is then lowered so that the earlier printed layer is covered by the same thickness of the liquid monomer and UV irradiation is applied. The layer-by-layer build process is repeated until the desired 3D part is achieved [29]. The printed part is only 80% cured at this point and the curing is further completed in a UV oven [13,30]. The schematic process of SLA system is presented in Figure 7. While SLA presents a high printing accuracy and smooth finishing, its disadvantages include high cost, time-consuming processing, brittle components and very limited choice of raw materials [16].

### 2.6. Inkjet Printing

**Figure 8:**
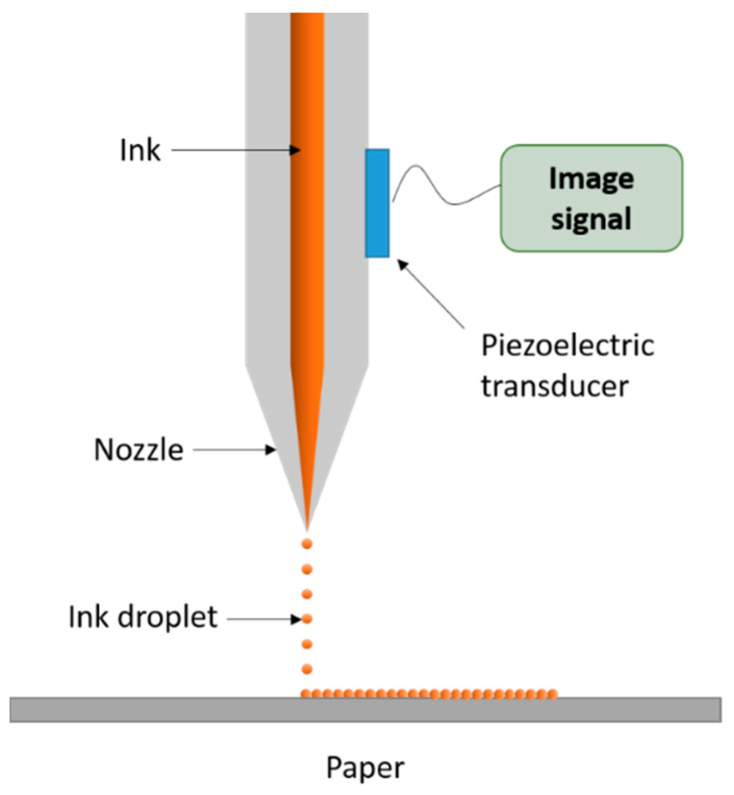
Schematic illustration of inkjet printing system.

Inkjet printing is the ideal AM technique for printing complex and advanced ceramic structures. This method involves the deposition of ceramic ink droplets onto the substrate through an injection nozzle. A continuous pattern, formed by the droplets, solidifies to a sufficient strength to support subsequent printed layers. The ceramic ink used is either a liquid suspension, which dries by the evaporation of the liquid or a wax-based ink, which is melted and deposited on the cold substrate to solidify [16]. The schematic process of inkjet system is presented in Figure 8.

While inkjet printing is a fast and efficient technique for printing complex structures, its drawbacks include coarse resolutions and poor adhesion between layers [16].

2.7. 4D Printing

4D printing is a newly developed field instigating from 3D printing [31]. It is defined as 3D printing + time, where the configuration, property, character, shape and/or functionality of the 3D printed object can change as a function of time with additional stimulation such as temperature [32], light [33], water [34] and pH [35]. The fundamental building blocks of 4D printing are 3D printing facility, stimulus, stimulus-responsive material, interaction mechanism and mathematical modelling [19].

## 3. Spectroscopy and Its Application in AM Techniques

The interaction of radiation with matter is utilised by spectroscopic analysis regimes to provide details of molecular energy levels, energy state lifetimes and transition probabilities of materials [81]. These details translate into information on the molecular environment and chemical structures of materials. 

While the term ‘spectroscopy’ refers to a number of techniques, the basic principle of all techniques is shining a beam of electromagnetic radiation onto a sample and observing how it responds to such a stimulus. Primarily, upon interaction with the sample, the radiation is either absorbed, reflected or scattered is some manner. The response is recorded as a function of radiation wavelength, resulting in a response plot (spectrum).

Different types of radiation, such as x-rays, ultra-violet and infrared, can be employed in spectroscopic characterisation of matter. X-ray photoelectron (XPS) and energy dispersive X-ray (EDXS) spectroscopies are typical material characterisation techniques which involve irradiation of a sample with X-rays. Fourier transform infrared (FTIR) spectroscopy involves absorption of infrared radiation of specific frequencies by molecules, dependent on their chemical structure. In ultraviolet—visible (UV) spectroscopy, atoms and molecules undergo electronic transitions when exposed to UV region of the electromagnetic spectrum. Similarly, Raman spectroscopy relies on inelastic scattering of the incident radiation. 

Material characterisation is crucial for fully realising the benefits of AM. While a lot of emphasis has been placed on characterisation of physical features of manufactured parts [14], the chemical properties warrant equal attention. Spectroscopic characterisation techniques offer chemical analysis regimes which help identify the material and determine elemental composition. This information, in turn, can be used to confirm material stability after processing, presence of functional materials within the manufactured parts and the purity of raw materials. Spectroscopic characterisation, thus can be useful for quality and process control. 

### 3.1. Spectroscopic Characterisations

#### 3.1.1. FTIR Spectroscopy

**Figure 9:**
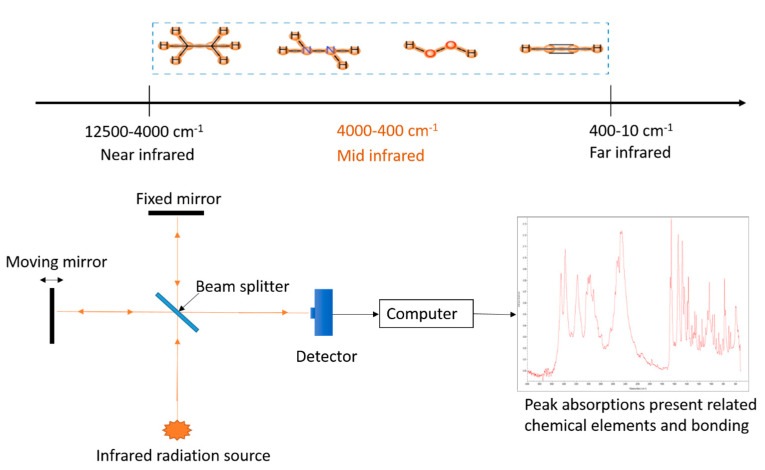
Working principle of Fourier-transform infrared spectroscopy (FTIR).

In FTIR spectroscopy, infrared radiation is applied to a sample and the transmission through the sample, or in some cases reflection from the sample is measured as a function of frequency (Figure 9). Chemical bonds between different elements absorb light at different frequencies, hence the materials’ absorbance of infrared light at various frequencies (wavelengths) helps determine the material’s chemical composition and structure [82]. FTIR spectroscopy is a cheap and fast technique with minimal sample preparation needs. On the downside, FTIR spectroscopy is water and carbondioxide sensitive and some compounds are difficult to analyse at low concentrations because of the interferences from other compounds. 

#### 3.1.2. Raman Spectroscopy

**Figure 10:**
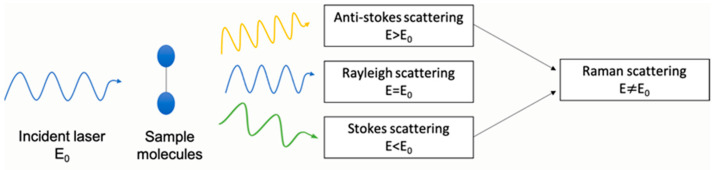
Working principle of Raman spectroscopy.

Raman spectroscopy is based on inelastic scattering of monochromatic light focussed on a molecular sample (Figure 10) [82]. Analysis of the scattered radiation from a molecular system indicates the presence of frequencies spectrally shifted to lower or higher energies compared to the incident radiation. The transfer of vibrational quanta between the interacting radiation and the medium results in spectrally shifted lines. Vibrational frequencies of functional groups in molecular systems can be characterised from the spacing of Raman lines. In the Raman spectrum, each bond is correlated to a specific energy [83]. Raman spectroscopy yields information complementary to IR absorption spectroscopy [81]. 

Raman spectroscopy is a non-contact and non-destructive technique capable of analysing chemical composition of materials. It typically requires no sample preparation and can be applied to different samples such as solids, powders, liquids, gels and slurries. Raman spectroscopy can also be combined with imaging facilities such as atomic force microscopy and confocal laser scanning microscopy. However, fluorescence of the sample can affect the Raman spectra and the intense laser irradiation can destroy the sample.

#### 3.1.3. UV-Vis Spectroscopy

**Figure 11:**
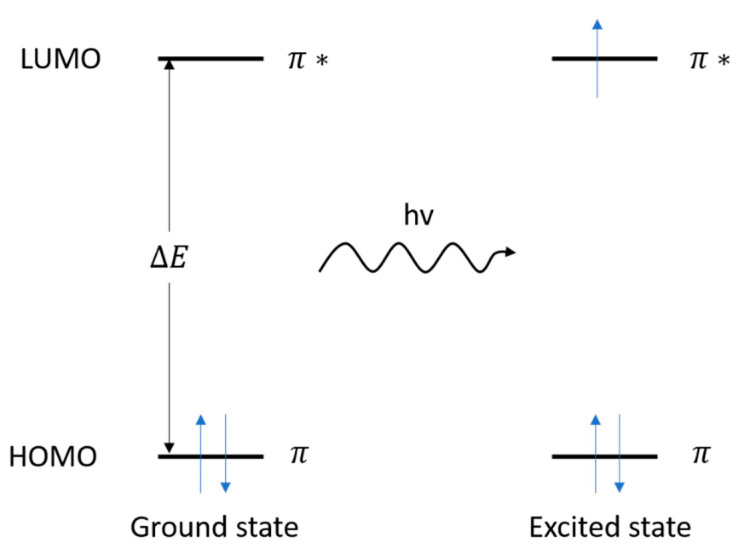
Working principle of UV-vis spectroscopy.

UV-vis spectroscopy involves the excitation of electrons from a low energy to high energy atomic or molecular orbitals when the material is irradiated with light from ultraviolet (100 to 400 nm) to visible region (400 to 800 nm) of the electromagnetic spectrum. Upon irradiation, a molecule absorbs a discrete quantity of energy to promote electrons to higher energy states, from the highest occupied molecular orbital (HOMO) to the lowest unoccupied molecular orbital (LUMO), resulting in an absorption spectrum with the intensity distribution of the spectrum reflecting the probabilities of the transitions [81] (Figure 11). While the UV-vis spectra are associated with the transitions between certain functional groups involving π and or n electron systems, they fall short of characterising the whole molecule. 

#### 3.1.4. XPS

**Figure 12:**
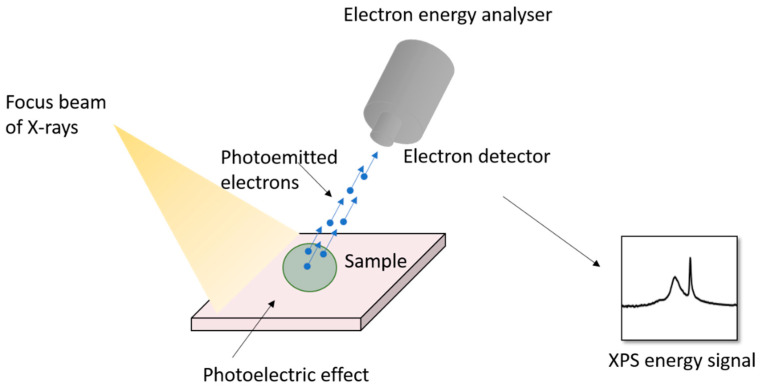
Working principle of X-ray photoelectron (XPS).

XPS has been widely used for measuring the elemental composition, speciation and electronic states of elements at the surface of samples [84]. In XPS, a solid sample is irradiated with soft X-rays to produce multiple ionisations from core and valence levels of the irradiated atoms or molecules [85] (Figure 12). The analyser measures the kinetic energy of the photoemitted electron and determines the corresponding binding energy, characteristic of a specific core level of the photoemitting atom [85]. While XPS provides detailed information on chemical bonding, it analyses only the surface of the sample (1–20 nm) and is an expensive technique employing sophisticated instrumentation and requires long processing time.

#### 3.1.5. EDXS

**Figure 13:**
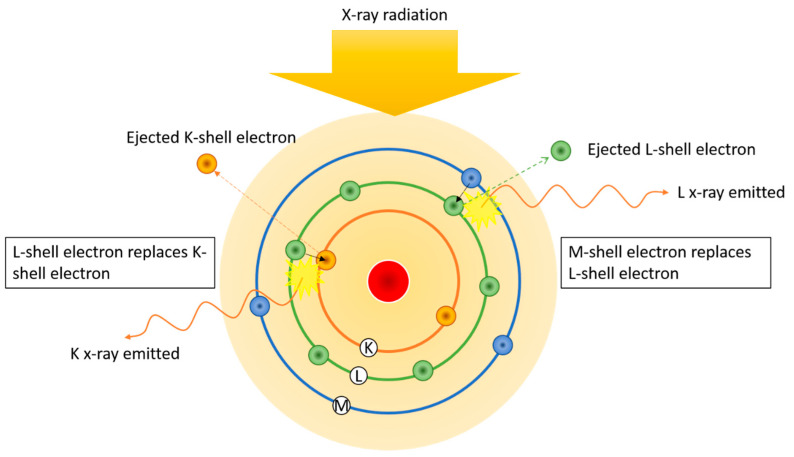
Working principle of energy dispersive X-ray (EDXS).

Like XPS, EDXS is a powerful and useful elemental analysis technique. It, however, has a higher sample analysis depth of 1–2 µm. EDXS is used in conjunction with scanning electron microscopy (SEM). In EDXS, a sample of interest is bombarded with a focussed beam of electrons which can excite and eject an inner shell electron, the vacancy being filled by an outer electron. An X-ray is emitted due to the energy difference between the two shells (Figure 13). The energies and number of emitted X-rays are measured by the spectrometer and are used to determine the elemental composition of the sample as the X-ray energies are characteristic of the atomic structure of the emitting element. While relative compositions (atomic %) and distribution maps of elements in the SEM scan area can be obtained using EDXS, the data generated is only for the surface to a depth of a few microns and not of the bulk. 

### 3.2. Applications of Spectroscopy 

#### 3.2.1. Biomedical Applications

With the fast development of AM techniques, their applications in the biomedical field have been contributing to an evaluable market share and trending to be the drivers for AM evolution and growth [16]. AM techniques provide important benefits for biomedical applications, such as high complex structures, customisation and patient-tailored properties, low-cost and fast prototype development, biocompatibility and good mechanical strength. 

**Figure 14:**
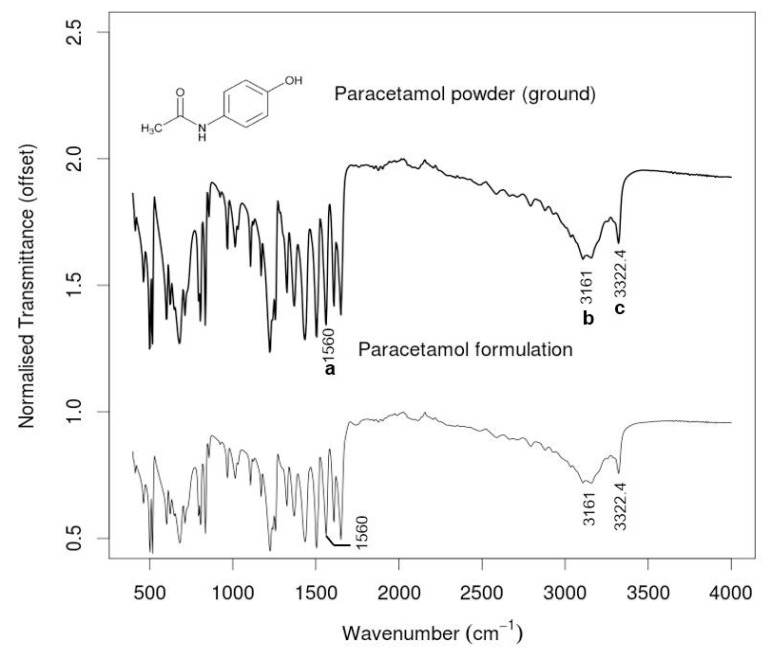
FTIR spectra of paracetamol powder and paracetamol formulation (a=N–H secondary amide, b=O–H, c=N–H secondary amide). Reproduced from [86], with permission from Elsevier.

Additive manufacturing is being increasingly utilised in drug-delivery applications and FTIR spectroscopy has emerged as a powerful tool to evaluate the stability as well as the interactions between the host matrices and the loaded drugs. Long et al. [41] have found that the carbonyl absorbance band of polylactic acid at 1756 cm^−1^ red shifted to 1735 cm^−1^ when progesterone was incorporated into the matrix via FDM. This shift was attributed to the possible interactions between polylactic acid and progesterone. The chemical structures of the polymer and the drug, however, remained largely unaffected as the spectral features of the 3D printed projectile were same as those of the raw materials. FTIR studies have also confirmed the absence of any detectable interactions between paracetamol and the excipients (poly (vinyl pyrrolidone) and croscarmellose sodium) in 3D printed immediate release tablets as the spectral features remained unchanged from paracetamol powder to the formulations (see Figure 14) [86]. FTIR spectrum of a 3D inkjet printed poly (ethylene glycol diacrylate) tablet containing ropinirole hydrochloride, collected using a spectrometer coupled with attenuated total reflectance attachment, was used to evaluate the degree of cure on the tablet surface [87]. The uncured poly (ethylene glycol diacrylate) monomer exhibited acrylate peaks at 1722 cm^−1^, 1636 cm^−1^, 1618 cm^−1^ and 810 cm^−1^, which were absent from the spectra of the cured tablet, indicating a high degree of acrylate conversion. 

**Figure 15:**
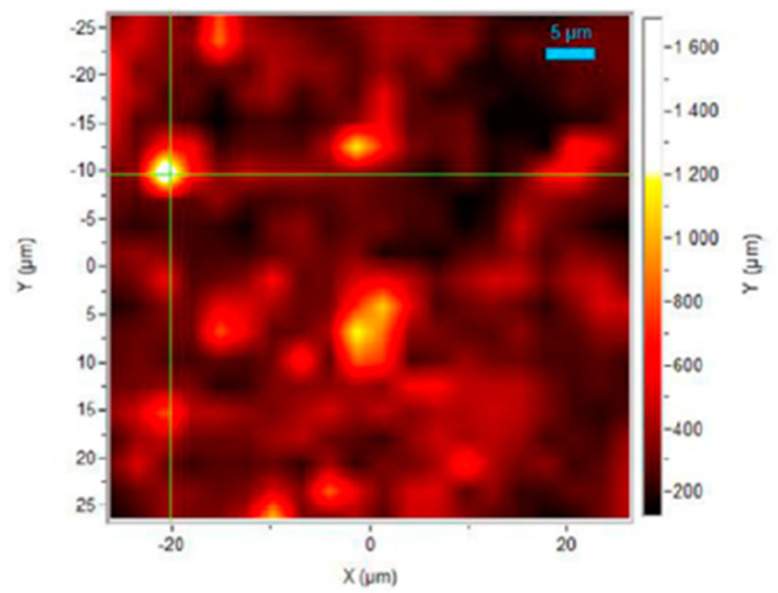
Raman intensity mapping of ropinirole hydrochloride in 3D printed tablet. Reproduced from [87], with permission from Elsevier.

The presence of nitrofurantoin anhydrate crystals in 3D printed antimicrobial eluting polylactic acid disks were confirmed by Raman spectroscopy [88]. Kollamaran et al. [36] have achieved low melting and thermolabile drug printlets, exhibiting 100% drug release within 20–30 min, at reduced FDM-operating temperature. The stability of thermolabile drugs over the 3D processing temperature has also been investigated using Raman spectroscopy. Significant differences in the Raman spectra of virgin and processed Rampiril indicated degradation of the drug at high temperatures. The low processing temperature for Ramipril was therefore, emphasised with the aid of Raman spectroscopy [36]. Using a hot melt 3D inkjet printing and beeswax as the drug carrier for fenofibrate, Kyobula et al. [89] fabricated personalised tablets with adjustable honeycomb cell size to control the drug release profiles. Raman spectrum, of the printed beeswax-fenofibrate mixture, showed no significant shifts in the position of the component bands in the mixture relative to the isolated components, indicating little to no structural changes to either beeswax or fenofibrate upon mixing and subsequent printing [89]. Moreover, the distribution of ropinirole hydrochloride in 3D printed crosslinked poly (ethylene glycol diacrylate) tablets has been studied by mapping the drug in the matrix using the drug related peak at 480 cm^−1^ (see Figure 15) [87]. While the confocal Raman mapping indicated the presence of the drug throughout the matrix, certain drug-rich ‘hot spot’ regions were also identified. 

With respect to tissue engineering, Santos et al. [90] improved the mechanical strength of 3D printed β-tricalcium phosphate scaffolds via a sintering process. The hypothesis that the sintering temperature affected the composition of the scaffolds was negated by FTIR evaluation of β-tricalcium phosphate powder. This conclusion was based on the characteristic phosphate group vibration bands at 1020 cm^−1^ and 962 cm^−1^ remaining relatively unchanged after the sintering process. FTIR spectroscopy was used again, in a later study on 3D printed polycaprolactone/β-tricalcium phosphate, to justify that the high extrusion temperature did not result in either chemical transformation or degradation of the polymer chains [91]. This was evident as the carbonyl group vibration band at 1727 cm^−1^ remained largely unaffected by the processing conditions. 

The presence of hydroxyapatite in 3D printed polylactic acid/nanohydroxyapatite bone substitute composite material was confirmed by the presence of calcium and phosphorous within the matrix by EDXS [37]. EDXS mapping further indicated that the nanohydroxyapatite particles were evenly dispersed in the polymer matrix even though the nanoparticles tend to agglomerate. EDXS elemental analysis focused on distinguishing cell boundaries from the interior of the cells have been performed on powder bed fusion 3D printed 316L stainless steel, which is widely employed in pharmaceutical manufacturing and medical implants such as cardiovascular implants (stents, artificial valves), orthopaedic bone fixation devices, orthodontic wires, plates and screws used in craniofacial applications and artificial eardrums [92]. Molybdenum enrichment was observed at the intercellular regions compared to the cell interiors and this had a significant impact on the 3D printed stainless steel. Similarly, EDXS mapping studies by Taylor et al. [93] have also presented high niobium presence at the bond regions between nickel titanium particles. Niobium powder had been added to the nickel titanium inks to form a eutectic nickel—titanium—niobium liquid phase during sintering while 3D printing. The eutectic phase bonded the nickel—titanium powders and improved the densification of the printed trusses.

The advances in AM techniques have been further applied in dentistry [94,95]. Recognizing the significant incidence of fungal infections in dental prostheses, functionalised dentures loaded with an antifungal agent were fabricated via the fused filament process. Functionalised dentures were 3D printed using polymethylmethacrylate filaments incorporated with polycaprolactone microspheres containing an antifungal agent. While the flexural strength of the dentures were characterised by mechanical testing, FTIR spectroscopy was used to confirm the presence of the antifungal agent in the printed dentures. Obvious peaks at 1017 cm^−1^ in the spectra of the printed dentures correlated with the C-C-H groups of the antifungal groups [96].

Furthermore, additively manufactured titanium alloys have been considered in the field of biomedical engineering [97,98], however, the selective laser melting process introduces high surface roughness due to partially melted particles which limits their use as hip implants [99]. Ghosh et al. [99] have therefore, modified the surface of the fabricated titanium implants with 2-methacryloyoxyethyl phosphorylcholine polymer. It was crucial to achieve a homogenous graft of the polymer on the implant surface, to minimise the friction and wear impact. The presence of the polymer grafts were hence, confirmed by FTIR spectroscopy, with –N^+^(CH_3_)_3_ and P=O bands attributed to phosphorylcholine units of the polymer.

#### 3.2.2. Electronics

Hu et al. [100] have used Raman spectroscopy to validate that graphene had been prevented from oxidation during laser additive manufacturing of graphene-copper nanocomposites, providing an alternative material for electrical and thermal conductors with strong mechanical properties. The Raman spectra of the nanocomposites exhibited peaks at 1354 cm^−1^, 1589 cm^−1^, 2697 cm^−1^, 1334 cm^−1^, 1574 cm^−1^ and 2677 cm^−1^, corresponding to multilayer graphene. Although the distinct peak shapes and positions showed that graphene had survived the fabrication process, its structure had changed slightly. Similarly, Raman spectroscopy has also been used to verify the existence of graphene sheets in 3D printed graphene—aluminium nanocomposites [84]. 

**Figure 16:**
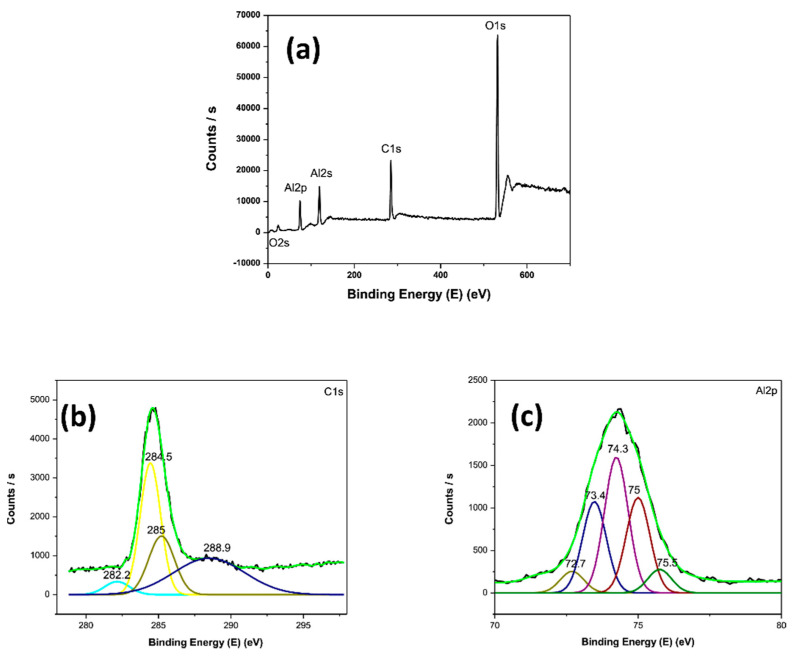
The XPS spectra of the laser 3D printed Gr-Al composites, (**a**) the whole survey XPS spectra, (**b**) carbon spectrum (**c**) Al spectrum. Reproduced from [84], with permission from Elsevier.

XPS performed on 3D printed graphene—aluminium nanocomposites indicated the sample surface, as expected, consisted of mainly carbon, aluminium and oxygen, as illustrated in Figure 16 [84]. The oxygen (O 1s) peak, indexed at 531 eV, may have been due to slight oxidation of the metal powders before printing. Further, the high resolution carbon and aluminium spectra showed peaks attributed to –COOH- groups and Al_2_O_3_. These results confirmed elemental composition of graphene—aluminium nanocomposites and identified the oxidation of the metallic powder. Considering unexpected oxidation widely existed in metal powder, proper and timely detection of oxidised raw material in pre-processing step could effectively prevent composition or quality issues in the following fabrication and post-processing stages. 

#### 3.2.3. Aerospace Applications

Most aerospace components have complicated structures with tailored geometries, leading to time-consuming and expensive manufacture processes [101]. Therefore, 3D printing is highly suitable for developing aerospace components, such as engine exhaust and turbine blade [102,103]. 

**Figure 17:**
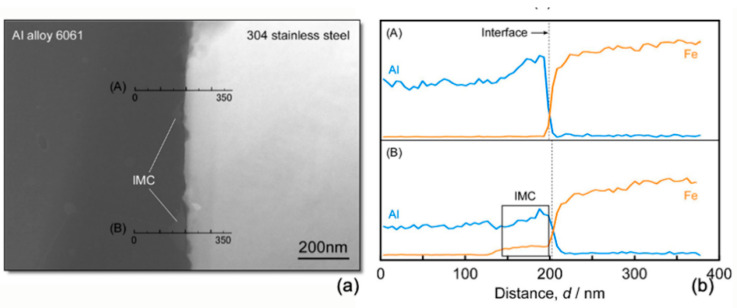
(**a**) Transmission electron microscopy bright-field image around the weld interface between Al alloy 6061 and 304 stainless steel; (**b**) the composition profile obtained by EDXS analysis. Reproduced from [98], with permission from Elsevier.

Taking advantages of ultrasonic crushing and super-cooled nucleation, ultrasonic assisted LENS can decrease the eutectic spacing and improve the fracture toughness of eutectic ceramic significantly [104]. While some hemispherical particles were observed on the Al alloy side of the weld interface in transmission electron microscopy (Figure 17a), EDXS was used to confirm the particles of Fe–Al intermetallic compounds (Figure 17b). 

Alumina (Al_2_O_3_) ceramics have been widely used as structural materials in aerospace. FTIR spectroscopy was also used to identify hydrophobic structures by chemisorption on alumina powder used for manufacturing micro-components by SLA [104]. The spectral features presented –(Si–O)_n_– vibrational bands at wavenumbers ranging from 1000 cm^−1^ to 1098 cm^−1^, indicating the silane coupling agents interacting with –OH groups on alumina surfaces. EDXS has also been used to confirm the presence and even distribution of aluminium oxide particles in UV curable acrylic-based resin suitable for SLA 3D printing of micro-components with complex geometries [105].

#### 3.2.4. Protective Applications

Compared to conventional protective structures, such as heavy and expensive solid monolithic plates made of high-strength steel or aluminium, AM brings possibilities to fabricate smart materials with superior thermal and sound insulation, better energy absorption and higher surface area [100]. Meanwhile the lightweight structures possess high stiffness-to-weight and high strength-to-weight ratios [16]. Thus, protective structures produced by AM makes a great fit for numerous engineering applications.

**Figure 18:**
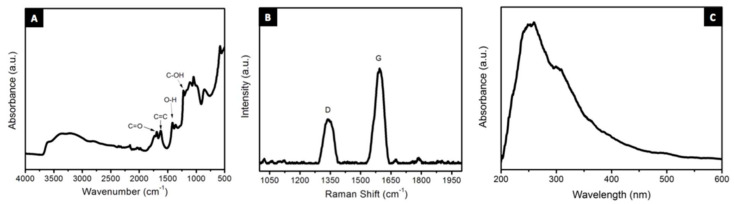
(**A**) FTIR, (**B**) Raman and (**C**) UV-Vis spectra of as-synthesised GO. Reproduced from [101], with permission from American Chemical Society.

Chemical modification using chlorosulfonic acid was carried out on 3D printed polystyrene objects to yield hydrophilic surfaces [106]. FTIR spectra was used to verify the chemical structure of the modified surfaces, with strong bands at 1370 cm^−1^ and 1170 cm^−1^ associated with O=S=O vibrations. Manapat et al. [107] have characterised graphene oxide powder used for 3D printing of high-strength nanocomposites. A 1 mg/mL sample was prepared by ultrasonicating the synthesised graphene oxide powder in water for 10 min and the absorbance was recorded using a StellarNet UV- vis near IR system. The recorded UV- vis spectra, presented in Figure 18, showed characteristic peaks at 230 nm and 300 nm; attributed to π–π* transitions of C=C in amorphous carbon systems and n–π* transition of C=O respectively. The conversion of graphene to its oxide form was thus confirmed by UV- vis spectroscopy, with additional information from FTIR and Raman spectroscopy (Figure 18). The surface oxygenated functional groups of graphene oxide were instrumental in forming hydrogen bonds with the host resins, resulting in 3D printed high strength nanocomposites. This high strength SLA 3D printed nanocomposites have been also characterised by Raman spectroscopy [108]. The mechanical properties of the conventional 3D resins were enhanced by graphene oxide fillers. While the graphene oxide particles formed hydrogen bonds with the oxygenated groups of the resins, it was difficult to ascertain the presence of graphene oxide in the nanocomposites thorough FTIR as many peaks overlapped with those of the resins. Raman spectroscopy was instead used in this study as the signature bands of graphene oxide at 1346 cm^−1^ and 1600 cm^−1^ were identifiable in the spectra. Raman spectroscopy was used further to verify the effect of temperature on the defect density of the nanocomposites. The number of point defects, calculated using the integrated intensity values from Raman spectra, were technically the same for the control and annealed samples, suggesting that the lower initial decomposition temperature of samples annealed at lower temperatures were not due to them having more defects. 

The XPS sample analysis depth ranges from 3–10 nm, making it an ultimate surface analytical technique [85]. O’Connor et al. [108] have determined the surface composition of 3D printed polyamide parts, produced by powder bed fusion, using XPS. The wide survey spectra presented peaks at 89, 154, 285, 350, 400, 532, 690 and 1072 eV, which correspond to magnesium, silicon, carbon, calcium, nitrogen, oxygen, fluorine and sodium respectively.

#### 3.2.5. 4D Printing Applications

The development of 4D printing opens a new door to shape-shifting structures, which makes 3D printing alive. Different AM techniques have been involved in creating 4D printing structures, with various stimulation-responded properties. 

**Figure 19:**
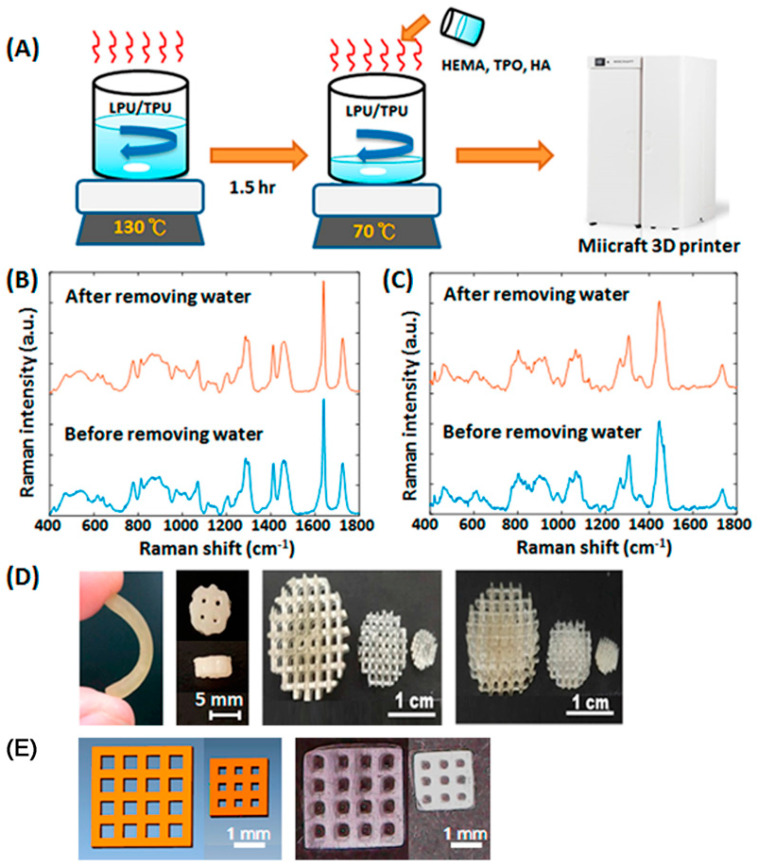
(**A**) The manufacturing process of the water-based polyurethane-based photosensitive materials; Raman spectra of the (**B**) water-based light-cured polyurethanes and (**C**) water-based thermoplastic polyurethanes with or without water removal processes; (**D**,**E**) The images printed structures. Reproduced from [80].

A light-cured 3D printed customised, polyurethane based, cartilage scaffold with shape-memory was reported by Shie et al. [80]. Raman spectra, Figure 19, showed no difference in the water-based polyurethanes between with or without water removing processes. Raman spectroscopy was also used to identify hyaluronic acid in the polyurethane matrix in this study. 

**Figure 20:**
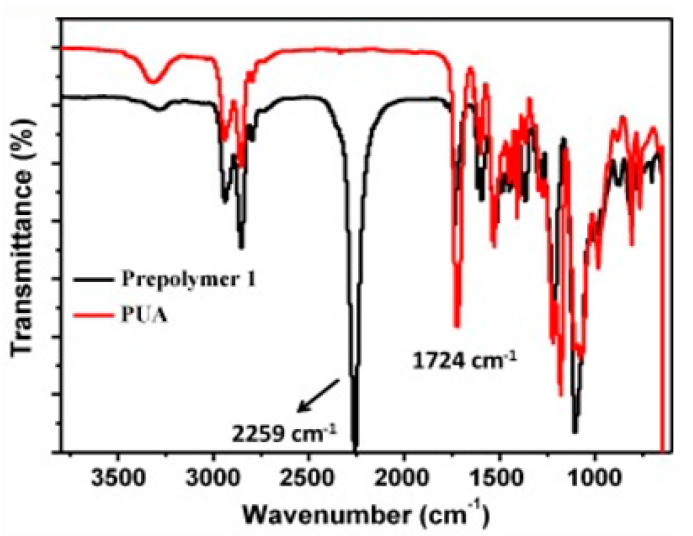
FTIR spectra of the prepolymer and polyurethane acrylate (PUA) measured in the synthetic process. Reproduced from [66], with permission from Elsevier.

Zhao et al. [66] have achieved a high printing accuracy of shape memory polyurethane processed by SLA. The high printing accuracy was attributed to the high UV activity of polyurethane acrylate compounded with epoxy acrylate and isobornyl acrylate as well as a radical photoinitiator. It takes less than 20 s for the deformed samples to recover their original shape in hot water baths, proving the high recovery rate. FTIR measurements were applied to monitor the synthetic process of polyurethane acrylate. The polymerisation process finished when the characteristic absorption peak of isocyanate groups (NCO) at 2259 cm^−1^, prominent in the prepolymer, disappeared while the absorption peak of carbonyl groups on the synthesised polyurethane at 1724 cm^−1^ reached the maximum value [66], as depicted in Figure 20.

**Figure 21:**
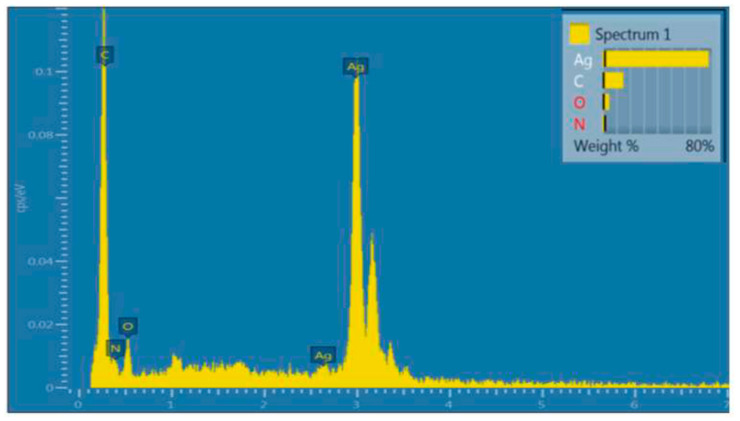
EDXS analysis indicating presence of silver on the surface of silver-functionalised 3D-printed poly(2-vinylpyridine) (P2VP). Reproduced from [35], with permission from American Chemical Society.

Nadgorny et al. [35] developed a pH-responsive material of poly(2-vinylpyridine) (P2VP) incorporated with silver nanoparticles for FDM, to print recyclable catalytic objects with dynamic and reversible pH-dependent swelling performance. The material shows a globule-to-coil transition upon protonation below pH 4.0, which makes it practical for the applications such as pH-responsive membranes and photonic-gels. FTIR was used to identify the formation of the characteristic pyridinium band through a band at 1639 cm^−1^. The catalysed reduction of 4-nitrophenol was identified by UV-vis through the conversion of 4-nitrophenol to 4-aminophenol. EDXS was also used to detect the surface of printed object is densely covered with silver (77.72 wt %, 29.45 atom %, see Figure 21).

Kuang et al. [79] reported a novel PCL-based ink of highly stretchable, shape memory (SM) and self-healing for UV-light-assisted direct-ink-write printing. The material can be stretched by up to 600% and showed high strain shape-memory and shape-memory-assisted self-healing capability, which has great potential for biomedical applications such as vascular repair devices. FTIR was used to confirm the polymerisation, showing the complete disappearance of vinyl characteristic peaks, such as the band of vinyl carbon–carbon double bond vibration at 1639 cm^–1^ [79].

Shiblee et al. [20] presented a shape-memory-hydrogel-based bilayer hydrogel actuator that can morph its shape in response to swelling in water, with temperature as an adjustable parameter. The bilayer structure was made of poly(N,N-dimethyl acrylamide-co-stearyl acrylate) with different concentrations of the crystalline monomer stearyl acrylate that responded to swelling-introduced stimulation with reversible shape-memory properties. The system is designed as a flower architecture that can change its shape after immersing in water and an underwater 3D macroscopic soft gripper that can grab, transport, and release a guest substance are developed to demonstrate the applicability of these hydrogels in biomimetic actuators, encapsulating systems and soft robotics. FTIR was used to verify the characteristic peaks and the absorbencies for the alkylene groups in DMAAm at 1646 cm^−1^, SA at 1636 and 984 cm^−1^ and MBAA at 1657 cm^−1^ almost disappeared in the spectrum of the SMG hydrogel, which signified that the radical reaction successfully occurred via 3D printing [20]. 

## 4. Spectroscopy in AM Process Loop

**Figure 22:**
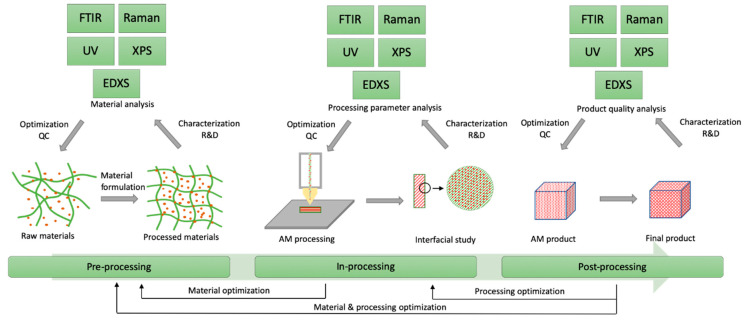
Spectroscopy in AM process-control loop.

Widespread application of AM is limited by product quality issues, such as material preparation and flaw, structure accuracy, undesired porosity, residual stresses, cracks or layer delamination, or microstructure inhomogeneity. Product quality issues can be attributed to raw materials preparation and AM processing parameters, which need to be processed by repeated and complex trial-and-optimisation cycles with inefficient, time-consuming and expensive outcomes. Process-control loop for AM has been attracting interests and were identified as measurement challenge vital for: monitoring of process and equipment performance, assurance of part adherence specifications and the ability to qualify and certify parts and processes [109]. Therefore, in situ monitoring of process and real-time control of AM process parameters find important applications to reduce variations and simplify optimisation steps in AM processes. In this consideration, spectroscopic techniques play essential roles in analysis and characterisation of AM materials and products, contributing to employment of better materials, increased production yield, reduced failure or rejection rate and enhancement of desirable qualities of final product. The application of spectroscopy in AM close-loop control system is illustrated in in Figure 22.

Spectroscopic techniques find their applications at each stage, which are summarised in Table 3 and demonstrated in the following section. 

Pre-processing control is attributed to material properties, such as the proper fusion, density, size distribution, particle morphology, chemical or physical interactions, viscosity and printability. Developing AM materials with proper characteristics can help define suitable processing parameters, ease of following in situ AM processing and improve the quality of final product. In pre-processing control, spectroscopic techniques offer promising evaluations such as elemental or molecular characters, functional bonding and absorption information of the feeding materials. 

In-processing control relates to two procedures: (i) Real-time monitoring: to monitor the properties of in situ product during AM processing, such as the interfacial geometry, layer lamination and adhesion, porous structure, flowability and printing accuracy; (ii) immediate feedback and regulation: based on the detected product properties especially errors and defects, feedback and regulation control can act immediately to improve the product quality by adjusting the processing parameters, such as pressure, temperature, laser strength, etc. Spectroscopic techniques combined with movable nozzle or heating parts of AM equipment can deliver a promising real-time measurement of the AM processing and provide valuable information for immediate feedback and regulation. In this consideration, fast-detecting and non-destructive techniques can be applied in real-time measurements. For instance, all AMs employ layer-by-layer building strategy, which may lead to discontinuity and inaccurate dimensions in all building directions [110]. In this case, Raman associated with imaging facilities such as atomic force microscopy and confocal laser scanning microscopy can offer real-time monitoring of being-built layer and then provide feedbacks to modify the AM building parameters to generate parts with better microstructure. However, in-processing control has been paid less attention than the pre- and post-processing controls, thus, there is an empty gap to be filled with proper applications and future development of spectroscopic techniques. 

Post-processing control aims to analyse the quality and characters of AM processed products, in order to evaluate if they meet final requirements or require material and/or processing optimisation(s). In this consideration, some key parameters of the processed product need to be examined, such as dimensional accuracy, surface roughness, porosity, mechanical properties, residual stress and fatigue strength [109]. In respect to post-processing, spectroscopic techniques offer valuable assessments in different aspects. For instance, FTIR can help evaluate the stability of materials in the form of detecting any chemical bonding changes as well as the interactions between the host matrices and the additive. In post-processing control, the assessments do not provide real-time investigation, however, more detailed information of the processing parameters can be obtained to optimise the AM process and improve the material issues, achieving desired performance of the final AM product.

## 5. Summary and Outlook

AM technologies have brought new possibilities and contributed to transforming the practice of biomedicines, electronics, aerospace, protective structures and the emerging 4D printing. The rapid progress in AM technologies has provided ease of material processing and cost and time savings for material manufacturing industries. It has also helped mitigate industrial challenges such as worker safety in harsh environments, decreased workforce availability and material wastage [15]. AM has thus created a niche in the material processing world. Additive manufacturing techniques such as FDM, LOM, SLA, SLS, SLM, Lens and inject printing have proven to be popular in the manufacturing world, and so have been other metal additive manufacturing process such as ultrasonic and friction stir additive manufacturing [111,112].

The development of AM technologies necessitates pertinent physical and chemical characterisation of both employed and produced materials, for defect evaluation, quality control and process improvement purposes. Non-destructive techniques such as ultrasonic testing are highly capable of detecting physical defects in engineered materials [113]. Moreover, neutron diffraction has been applied for the evaluation of the microstructure of metal parts [114]. Although spectroscopic analysis may require cutting a small portion of the manufactured parts or sample preparation such as thinning, spectroscopy finds its usefulness in chemical characterisation of materials. Spectroscopic analysis can be applied for the characterisation of raw materials as well as manufactured products. The choice of spectroscopic techniques, however, would depend on the characterisation needs as well as the sample compatibility. For example, interference might occur if UV-vis spectroscopy was applied to UV cured parts. Besides, the spectroscopic methods might not be suitable to in-process monitoring for metal/alloy additive manufacturing because of strong heat-induced light, radiation or even plasma emission.

FTIR, UV-vis and Raman spectroscopies are conventional techniques which provide fundamental information on the functional groups of materials. EDXS provides a basic elemental scan of elemental composition of the material. XPS is a more advanced method for determining the elemental weight composition and oxidation states of the elements. Although most spectroscopic techniques are surface characterisation techniques, they are however, helpful in determining the presence of specific elements in the material, confirming the stability of materials after heat treatment during AM, compatibility of different material components and affirming the presence of functional materials in additively manufactured parts, such as antifungal properties in 3-D printed dentures [96].

The application of spectroscopy in AM continues to evolve and new applications keep emerging. Resonant ultrasound spectroscopy has been employed for detecting part to part microstructure variability between built AM components [115]. Laser-induced breakdown spectroscopy has provided real-time quantitative multi-elemental analysis during the manufacturing process [116,117]. Moreover, optical emission spectroscopy has been used for detecting structural features of metal alloys [118]. 

While spectroscopy has been applied in process controlling of parts growth by AM [116], the real-time sensor or program in in situ monitoring capability of spectroscopic techniques needs to be developed further. Consequently, the feedback mechanism after detecting the defects in AM process-control loop is inadequate. Future work could possibly focus on developing the in situ monitoring in spectroscopies and programming the mechanism to give in-time feedback so that corresponding reactive actions could be taken during the AM processes. 

## Figures and Tables

**Table 1:** Some successful commercial applications of AM.

Sector	Company/Institute	Year	AM technique and Materials	Development	Parts Function
Consumer goods	Holthinrichs Watches	2017	Laser powder bed fusion; stainless steel 316L.	Parts for limited edition watches [7].	3D printed case, crown and buckle.
Nuclear energy	US Department of Energy	2018	Dissolvable materials.	Nuclear plant components [8].	Dissolvable supports with improved topology and microstructure.
Manufacturing	Renishaw	2019	N/A	Parts for arm powered bike [9]	Central titanium support (CTS)
Manufacturing	Siemens	2019	N/A	Components for aeroderivative gas turbine [10].	Dry low emission (DLE) pre-mixer
Catalysts	Flemish Institute for Technological Research	2019	3D fibre deposition technique; Ni-alumina catalysts	Ni-alumina-based catalysts for CO_2_ methanation [11].	CO_2_ conversion and selectivity to methane

**Table 2:** Summary of AM techniques.

AM Techniques	Input Stock	Working Principle	Advantages	Disadvantages	Materials		Spectroscopic Characterisations
					Class	Specifications	
FDM	Solid	Thermal fusion; Nozzle extrusion deposition.	Low cost; High speed/	Limited to thermal-stable substances; Mechanical weakness; Rough appearance; Limited to thermoplastic materials.	Composite	Vinylpyrrolidone—vinyl acetate copolymers and polyvinyl pyrrolidone loaded with mannitol, ramipril and magnesium carbonate [36] Polylactic acid with nanohydroxyapatite [37] Polycaprolactone and polylactic acid composites [38] polylactic acid with graphene nanoplates/multi-walled carbon nanotubes [39] wood flour with thermoplastic polyurethane [40]	FTIR [38,40,41,42,43] Raman [36,39,42] XPS [40,42,44] EDXS [37,44]
					Polymer	Polylactic acid [42,45] Polycarbonate [46] poly (acrylonitrile butadiene styrene) [43] poly(ϵ-caprolactone) [44]	
Powder bed fusion	Powder	Laser sintering/fusion; Local in situ fusion; Material powder bed.	Wide materials choices; Good mechanical consolidation	Limited accuracy; Required gas atmosphere; Undesired porosity and balling; Residual stresses, Cracks or layer delamination; Microstructure inhomogeneity	Alloy	Titanium alloys (Ti-45Nb) [47] Titanium-zirconium-niobium [48] Cobalt-chromium-molybdenum [49]	FTIR [50,51,52] Raman [53,54] XPS [49,50,53] EDXS [47,48,50,54,55]
					Composite	Titanium alloy (Ti-6Al-4V) with poly (2-methacryloyloxyethyl phosphorylcholine) [50] Carbon nanotube/molybdenum-titanium-aluminium [54] Polyamide 12 coated with hydroxyapatite nanoparticles [51] Poly-ε-caprolactone and hydroxyapatite [52]	
					Polymer	Polyamide 12 [53]	
					Ceramic	Spodumene powder [55]	
LENS	Powder	Laser melting; Local powder feeding.	Repairing parts; Material graded deposition;	Stress residuals	Alloy	AlCoCrFeNi [56] Hydroxyapatite coating on titanium alloy (ti6al4v) [57] Titanium powder [58] Fe–Ti alloys [59]	FTIR [57] Raman [60] UV-vis spectroscopy [57] XPS [58] EDXS [56,59]
					Ceramic	Yttria stabilised zirconia [60]	
LOM	Solid	CO_2_ laser melting; Laminated layer building and adhesion.	Large object manufacture; low cost.	Low surface definition; challenge in complex internal structures.	Graphene	Laser-introduced graphene [61]	FTIR [62] Raman [61] XPS [61,63] EDXS [64,65]
					Alloy	Titanium alloy (Ti-6Al-4V) [63]	
					Ceramic	Titanium carbide—silicon carbide [65] Silicon nitride [64] Alumina [62]	
SLA	Liquid	Liquid monomers by UV polymerisation; laser beam solidification.	High printing accuracy and smooth finishing	High cost; time consuming processing; brittle components; very limited choice of raw materials (only UV-resistant)	Polymer	Polyurethane acrylate, epoxy acrylate, isobornyl acrylate [66] Perfluoropolyether [67] Resin [68,69]	FTIR [66,70,71] Raman [68] UV-vis spectroscopy [67,69] XPS [71] EDXS [72]
					Ceramic	Alumina [72] Silicon oxycarbide [70] Silicon nitride [71]	
Inkjet printing	Powder	Ceramic ink droplets; solidification by drying liquid layers.	Complex structures;	Expensive ink; limited in high volume manufacturing; low printing speed.	Polymer	Polyvinyl alcohol [73]	FTIR [73,74] Raman [74,75] XPS [76] EDXS [75,77]
					Ceramic	Alumina [77] Calcium sulphate hemihydrate [75]	
					Composite	Lactic acid and ethanol [74] Indium nitrate hydrate, gallium nitrate hydrate and zinc acetate dihydrate [76]	
4D printing	Solid	Using 3D printing facility and smart materials to achieve a stimulation-responded structure.	Self-assembly; reconfiguration; transformation (save space for storage/transport); multi-functionality; self-repairing.	Based on the 3D printing techniques involved.	Temperature-responsive	Soybean oil epoxidised acrylate [78] PCL-based resin [79]	FTIR [20,35,66,78,79] UV-vis spectroscopy [35] Raman [80] EDXS [35]
					Light-responsive	Polyurethane acrylate with epoxy acrylate, isobornyl acrylate and radical photoinitiator [66] Polyurethane [80]	
					Water-responsive	Poly(N,N-dimethyl acrylamide-co-stearyl acrylate) [20]	
					pH-responsive	Poly(2-vinylpyridine) with addition of 12% acrylonitrile−butadiene−styrene (ABS) [35]	

**Table 3:** Spectroscopic characterisations in AM process-control loop.

Spectroscopy	Wave Propagation Principle	Effective Penetration Depth	Materials Analysed	Sample Preparation	AM Process-Control Loop	Spectroscopy Function	Diagnosis by Spectroscopy in AM Process
FTIR	Refraction	0.5–3 µm	Polymers, ceramics	FTIR spectrometer equipped with attenuated total reflectance accessory is capable of analyzing solids and requires minimal sample preparation. A small piece of specimen is placed directly on the stage for analysis.	Pre-processing	Elemental, molecular characters and functional bonding; predict potential reactions.	Raw material blending; additive homogeneity.
					In-processing	Stability or chemical bonding changes.	Proper temperature control; degradation of feeding materials.
					Post-processing	Interactions between materials; phase solubility/precipitation.	Degradation or changes in materials properties; blending uniformity.
Raman	Diffraction	0.2–10 µm	Polymers, ceramics	Sample areas from 200 × 200 µm^2^ up to 50 × 50 mm^2^ can be scanned. Essentially no sample preparation is required. A small piece of specimen is placed of the sample stage for analysis.	Pre-processing	Molecular and bonding.	Raw material blending; additive homogeneity.
					In-processing	Microstructure, gains, cracks, imperfections.	Influence of local heating; interfacial interaction between layer to layer; structural defect.
					Post-processing	Stability, existence/distribution of additives.	Degradation or changes in materials properties; dispersion guidelines.
UV-vis spectroscopy	Refraction	1–10 µm	Polymers, ceramics	Thin transparent films can be directly analyzed in the transmission mode. Diffuse reflectance mode can be employed for solid samples. Powders are diluted with a barium sulfate standard	Pre-processing	Elemental information	Raw material selection.
					In-processing	Stability or chemical bonding changes.	Degradation or changes in materials properties; bonding development or disruption during fusion.
					Post-processing	Chemical or bonding changes	Materials property evaluation; absorbance assessment; functionality.
XPS	Diffraction	1–10 nm	Polymers, ceramics, metals	Minimal sample preparation is required. Powders are pressed into clean indium foils. Solid samples are secured on the sample holder using carbon tapes.	Pre-processing	Elemental and molecular characters, chemical bonding.	Raw material selection and blending; addictive homogeneity.
					In-processing	Chemical changes in materials	Degradation/changes in materials properties; influence of local heating on materials.
					Post-processing	Chemical changes; molecular bonding changes.	Degradation/changes in materials properties; metal oxidation.
EDXS	Diffraction	1–2 µm	Polymers, ceramics, metals	Small pieces fo solid samples are secured on the sample stubs with carbon tapes. Non-conductive samples are usually coated with gold or platinum particles.	Pre-processing	Elemental and molecular characters, chemical bonding.	Dispersion guidelines; material blending and selection assessment.
					In-processing	Bond regions, distribution of elements.	Influence of local heating; interfacial interaction between layer to layer; interlayer adhesion; Insufficient fusion or melt; flow rate control.
					Post-processing	Elemental composition, atom structure, distribution map.	Porosity or structural defect; functionality; dispersion assessment.

## Data Availability

No new data were created or analyzed in this study. Data sharing is not applicable to this article.
